# Performance Investigation of Proteomic Identification by HCD/CID Fragmentations in Combination with High/Low-Resolution Detectors on a Tribrid, High-Field Orbitrap Instrument

**DOI:** 10.1371/journal.pone.0160160

**Published:** 2016-07-29

**Authors:** Chengjian Tu, Jun Li, Shichen Shen, Quanhu Sheng, Yu Shyr, Jun Qu

**Affiliations:** 1 Department of Pharmaceutical Sciences, University at Buffalo, State University of New York, Buffalo, United States of America; 2 New York State Center of Excellence in Bioinformatics and Life Sciences, 701 Ellicott Street, Buffalo, United States of America; 3 Center for Quantitative Sciences, Vanderbilt University School of Medicine, Nashville, United States of America; Indiana University, UNITED STATES

## Abstract

The recently-introduced Orbitrap Fusion mass spectrometry permits various types of MS2 acquisition methods. To date, these different MS2 strategies and the optimal data interpretation approach for each have not been adequately evaluated. This study comprehensively investigated the four MS2 strategies: HCD-OT (higher-energy-collisional-dissociation with Orbitrap detection), HCD-IT (HCD with ion trap, IT), CID-IT (collision-induced-dissociation with IT) and CID-OT on Orbitrap Fusion. To achieve extensive comparison and identify the optimal data interpretation method for each technique, several search engines (SEQUEST and Mascot) and post-processing methods (score-based, PeptideProphet, and Percolator) were assessed for all techniques for the analysis of a human cell proteome. It was found that divergent conclusions could be made from the same dataset when different data interpretation approaches were used and therefore requiring a relatively fair comparison among techniques. Percolator was chosen for comparison of techniques because it performs the best among all search engines and MS2 strategies. For the analysis of human cell proteome using individual MS2 strategies, the highest number of identifications was achieved by HCD-OT, followed by HCD-IT and CID-IT. Based on these results, we concluded that a relatively fair platform for data interpretation is necessary to avoid divergent conclusions from the same dataset, and HCD-OT and HCD-IT may be preferable for protein/peptide identification using Orbitrap Fusion.

## Introduction

High resolution mass spectrometers play a pivotal role in the field of proteomics and recent improvements in sensitivity and scan rates enable them to detect ~90% of the expressed proteome for yeast[1]. The traditional collision induced dissociation (CID, or resonant excitation CID) and higher energy collisional dissociation (HCD, or beam-type CID) are the most popular fragmentation techniques for bottom-up proteomics studies[2, 3]. Tandem mass spectra (MS2) by CID with the detection of ion trap (CID-IT) and HCD with the Orbitrap detection (HCD-OT) are the two common MS2 acquisition methods, presenting the advantages of speed and sensitivity for the former and wide m/z range and accurate MS2 for the latter[2–4]. The comparative studies of HCD-OT and CID-IT have been investigated and divergent conclusions have been presented from different labs[2, 5–7], even for the same data set[2, 7]. This may be caused by the different parameters of MS and different MS data interpretation methods they have used. Moreover, the new MS instrument Orbitrap Fusion enables MS2 fragmentation by HCD with IT detection (HCD-IT)[1], combining the benefits of wide range of m/z products and speed. However, it is not clear which MS2 acquisition method provides better sensitivity, reproducibility and convenience for proteomics.

With the rapid development of MS instruments, database search engines such as SEQUEST[8], Mascot[9], OMSSA[10], MyriMatch[11], Andromeda[12], Morpheus[13], MS-GF+[14], and MS Amanda[15] are subsequently developed to interpret the different types of spectra generated by MS. To achieve confident and accurate MS-based identification, the target-decoy search strategy is widely applied because of its conceptual simplicity and easy implementation[16]. Moreover, besides the utilization of original score to calculate false discovery rate (FDR), many post-search algorithms have been developed to perform statistical classification between correct and incorrect using machine learning methods[17–24] such as PeptideProphet[17, 24] and Percolator[20]. The Percolator algorithm[20] initially identifies a subset of high-confidence target PSMs, and then learns to optimally separate correct and incorrect PSMs using the support vector machines (SVM)-based classifier. More peptide/protein identifications are achieved using Percolator while compared to other filtering methods[20]. The different combinations of search engines and post-processing approaches may also give different conclusions for comparative analysis of the same data sets.

In this work, we evaluated different MS2 acquisition methods, including HCD-OT, HCD-IT and CID-IT and CID-OT on one of the most recent instruments, Orbitrap Fusion. To select a relatively fair platform for data interpretation, popular search engines (e.g. SEQUEST and Mascot) and filtering approaches (e.g. score-based, PeptideProphet, and Percolator) were applied to analyze all the raw files in this study. Sensitivity, reproducibility and convenience for large-scale data analysis of these MS2 acquisition methods were investigated and discussed. Based on these results, the optimal MS2 strategy and corresponding data interpretation approach could be suggested for other proteomics researchers to achieve better proteome coverage from the Orbitrap Fusion or similar instruments.

## Materials and Methods

### Sample preparation

The PANC-1 cells (human pancreatic carcinoma cell line) were from Dr. William J. Jusko’s lab in the Department of Pharmaceutical Sciences at University at Buffalo (Buffalo, USA). Cell samples were homogenized by ultra-sonication (Sonicatore XL-2000, Misonix, Inc., USA) in an ice-cold lysis buffer (50 mM Tris-formic acid, 150 mM NaCl, 0.5% sodium deoxycholate, 2% SDS, 2% NP-40 substitute, pH 8.0) for 3–5 second bursts, until the solution was clear. Lysates were centrifuged at 20,000 g for 30 min at 4°C. The resulting supernatant was transferred to a new tube and BCA Protein Assay (Pierce, Rockford, IL) was used for protein concentration determination. All samples were stored at -80°C until further analysis. For each sample, 100 ug of protein was reduced with 10 mM DTT for 30 min at 56°C, and then alkylated with 30 mM IAM for 30 min in darkness at 37°C. In this study a precipitation/on-pellet-digestion procedure was used to perform precipitation and tryptic digestion as previously described [25, 26].

### NanoLC-MS/MS analysis

The peptide mixture of PANC-1 cells was analyzed using an ultra-high pressure Ekspert nanoLC 425 system (Eksigent, Dublin, CA) coupled to a Orbitrap Fusion tribrid mass spectrometer (Thermo Fisher Scientific, San Jose, CA). The mobile phase consisted of 0.1% formic acid in 2% acetonitrile (A) and 0.1% formic acid in 88% acetonitrile (B). Each 4 μg of peptide mixture was loaded onto a reversed-phase trap (300 μm ID x 0.5 cm, packed with Zorbax 3-μm C18 particles), with 1% mobile phase B at a flow rate of 10 μL/min, and the trap was washed for 3 min. A series of nanoflow gradients (flow rate, 250 nL/min) was used to back-flush the trapped samples onto the nano-LC column (75 μm ID x 100 cm, packed with Pepmap 3-μm C18 particles) for separation. The nano-LC column was heated at 52°C to greatly improve both chromatographic resolution and reproducibility. A 160-min gradient was applied in these analyses to achieve sufficient peptide separation. The gradient profile was as follows: 0 to 3% B over 3 min; 3 to 6% B over 5 min; 6 to 28% B over 118 min; 28 to 50% B over 10 min; 50 to 97% B over 1 min; and finally isocratic at 97% B for 23 min, and then the column was equilibrated with mobile phase A.

**Fig 1:**
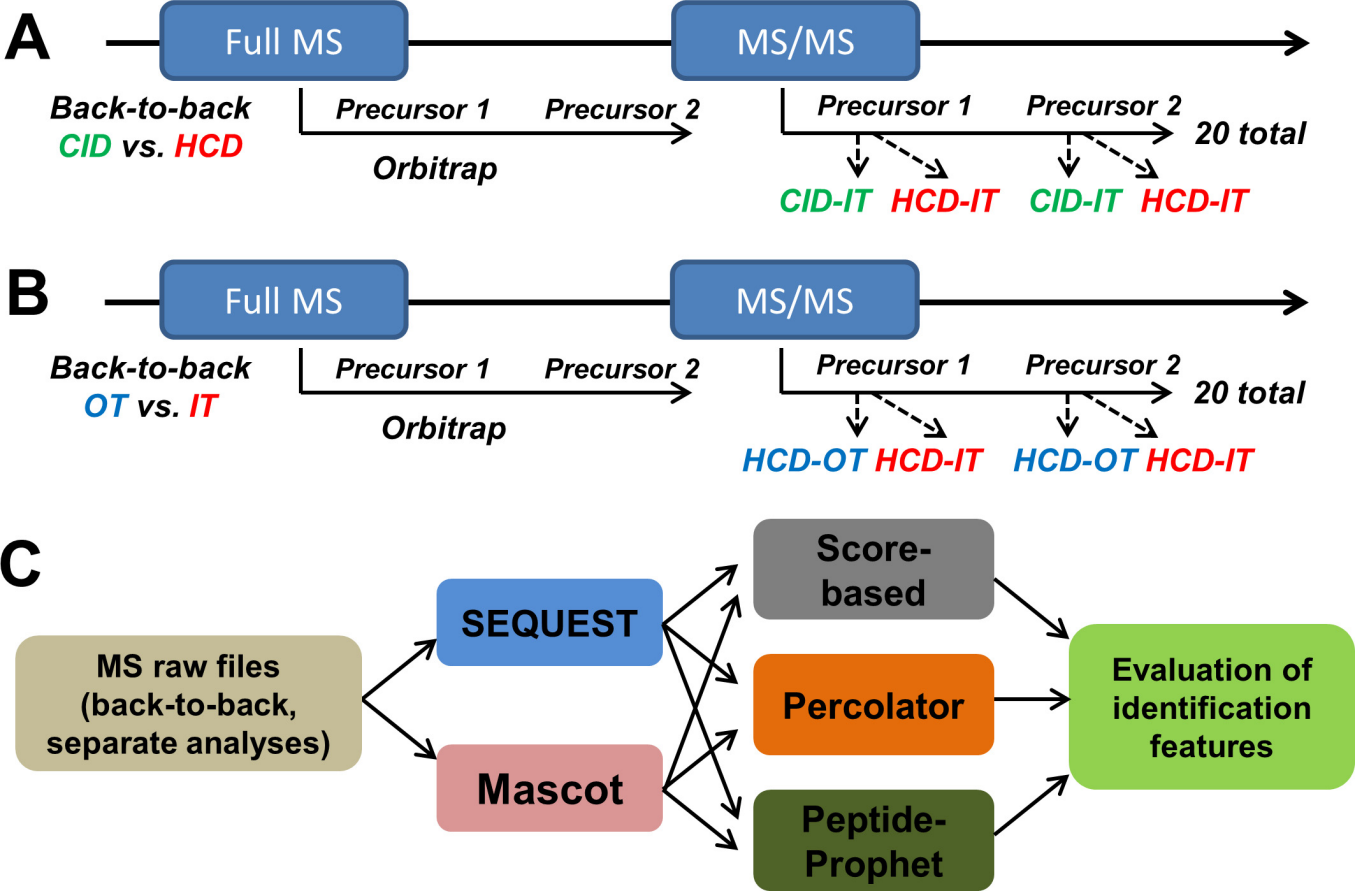
Flowchart of evaluation of CID- and HCD-type fragmentation, OT- and IT-type detection, and optimal MS2 acquisition method in the Orbitrap Fusion mass spectrometer. We used the back-to-back strategy to perform the comparisons: HCD *versus* CID, and OT *versus* IT. (A) The 20 most abundant ions from each full MS cycle in Orbitrap were subjected to sequential CID-IT (ion-trap detection) and HCD-IT. (B) The 20 most abundant ions from each full MS cycle in Orbitrap were subjected to sequential HCD-OT (Orbitrap detection) and HCD-IT. (C) The files of resulting back-to-back analyses and separate MS2 acquisition methods (HCD-OT, HCD-IT, CID-OT, CID-IT) were analyzed using different search engines (SEQUEST, Mascot) and post-processing approaches (Sore-based, Percolator, PeptideProphet).

The data-dependent product ion mode was applied for all analyses. For precursor peptides fragmentation and detection, MS1 survey scans (m/z 400 to 1500) were performed at a resolution of 120,000 with a 5 × 10^5^ AGC target. A resolution of 60,000 for MS1 was also investigated and a lower number of peptides was observed relative to a resolution of 120,000 we applied here (S1A Fig). Peptide precursors with charge state 2–7 were sampled for MS2. The instrument was run in top speed mode with a cycle time of 3 s. Dynamic exclusion was enabled with the following settings: repeat count = 1; exclusion duration = 60 s; mass tolerance = ± 10 ppm. Monoisotopic precursor selection was turned on. Tandem MS was performed by isolation at 1.0 Th with the quadrupole for HCD or CID fragmentation. The normalized collision energy (NCE) was 35% for both HCD and CID. Comparison between the NCE of 30% and 35% for HCD was performed, and we found that the use of 35% NCE for HCD gave similar or a little bit more protein identifications (S1B Fig). For OT detection, tandem mass spectra were analyzed with a resolution of 15,000, MS2 AGC target was set to 5 × 10^4^ and the max injection time was 50 ms. Maximum injection times of 20, 35 and 50 ms were also evaluated and the use of 50 ms gave more peptide/protein identifications (S1C Fig). For IT detection, tandem mass spectra were analyzed by ion trap with rapid scan rate, MS2 AGC target was set to 1 × 10^4^ and the max injection time was 35 ms. In back-to-back (B2B) analyses (Fig 1), the 20 most abundant ions (TOP20) detected in an Orbitrap full MS spectrum were selected for further MS/MS analyses. For comparison of CID-IT and HCD-IT, each precursor ion was first selected for CID-fragmentation with IT detection and then for HCD-fragmentation with IT detection; for comparison of HCD-OT and HCD-IT, each precursor ion was first selected for HCD-fragmentation with OT detection and then for HCD-fragmentation with IT detection. Other MS settings were the same as described above. Each type of MS2 strategies was analyzed in triplicate.

### Database search and post-search filtering analyses

Proteome Discoverer (PD, version 1.4.1.14, Thermo-Scientific) was used to perform database searching against Swiss-Prot human protein database (20,166 entries, released January, 2015). Search engines: SEQUEST-HT and Mascot (version 2.4.0) implemented in Proteome Discovery were individually applied for all MS raw files. The search parameters used were as follows: 20 ppm tolerance for precursor ion masses, 1.0 Da tolerance for fragment ion masses analyzed by ion trap, 0.02 Da tolerance for fragment ion masses analyzed by Orbitrap. We also evaluated 0.35 Da tolerance for fragment ion masses analyzed by HCD-IT as suggested by previous reports[27]. Lower peptide/protein identifications were achieved relative to the use of 1.0 Da tolerance when using SEQUEST-PeptideProphet as shown in S1D Fig and no difference was observed when using SEQUEST-Percolator as we previously studied [28]. Thus, 1.0 Da tolerance for fragment ion masses analyzed by ion trap was applied in this study as we previously reported[28]. Two missed cleavages were allowed for fully tryptic peptides. Carbamidomethylation of cysteines (+57 Da) was set as a fixed modification, and methionine oxidation (+16 Da) was set as a variable modification. The false discovery rate (FDR) was determined by using the target-decoy search strategy[29]. The sequence database contains each sequence in both forward and reversed orientations, enabling FDR calculation. Here, peptide/protein FDR is calculated as the number of decoy peptides/proteins divided by the number of target peptides/proteins. The FDR was set to 0.01 for both peptide and protein identifications.

For different post-search filtering approaches, Scaffold version 4.3.2 (Proteome Software, Portland, OR) and custom software BuildSummary (v7.1.1)[30] were used in this study. For score-based filtering, PSMs were sorted and selected according to scores to achieve a protein FDR of 1.00%: ascending XCorr (Delta Cn ≥ 0.1) for SEQUEST-HT and ascending ion score for Mascot; For PeptideProphet (with delta mass correction), ascending probability was used to select confident PSMs for SEQUEST-HT and Mascot; For Percolator, ascending SVM-score was used. The score threshold yielding the maximum target protein groups at less than or equal to 1% FDR was determined. Percolator (version 2.04), incorporated in PD, was used to generate q-values, SVM-scores and posterior error probabilities. The newest version of BuildSummary could be downloaded freely at https://github.com/shengqh/RCPA.Tools/releases/.

## Results and Discussion

To date, because of the prevalent use of hybrid MS, multiple MS2 acquisition methods are available on the same instrument, and suitable data interpretation approaches were correspondingly developed for maximizing the identifications. However, evaluation of different MS2 acquisition methods on Orbitrap Fusion using a relatively fair platform has not been comprehensively investigated. Because of the data-dependent MS2 analyses and different speed of HCD and CID acquisition, the back-to-back strategy is often performed for parallel comparison between the HCD-OT and CID-IT to ensure that identical precursors were respectively analyzed[2]. In previous studies, the mixed effect of HCD fragmentation and OT detection are commonly compared to CID-IT [2, 7], and more peptide-spectra-matches (PSMs) by HCD-OT than CID-IT is observed using the back-to-back strategy. Here we designed the back-to-back analyses of CID-IT *versus* HCD-IT, and HCD-OT *versus* HCD-IT to separately evaluate the different fragmentations (HCD *versus* CID) and detections (OT *versus* IT) for peptide identification on the Orbitrap Fusion mass spectrometer. The scheme of back-to-back experimental designs was shown in Fig 1A & 1B. Three LC-MS/MS replicates were analyzed for each comparison by two popular search engines (SEQUEST and Mascot) and three common filtering approaches (original score, PeptideProphet, and Percolator) as shown in Fig 1C. SEQUEST and Mascot are two classical search engines with different scoring strategies. SEQUEST utilizes a cross-correlation score to evaluate the similarity between experimental and theoretical mass spectra while Mascot emphasizes the rank of possible peptide matches that best fits the acquired MS2 via probabilistic modeling. The 1% of FDR at respective peptide level and protein level was applied in all analyses. Separate analyses of different MS2 acquisition methods (S2 Fig) were also investigated.

### Back-to-back comparison for HCD- and CID-type MS2 fragmentations

**Fig 2:**
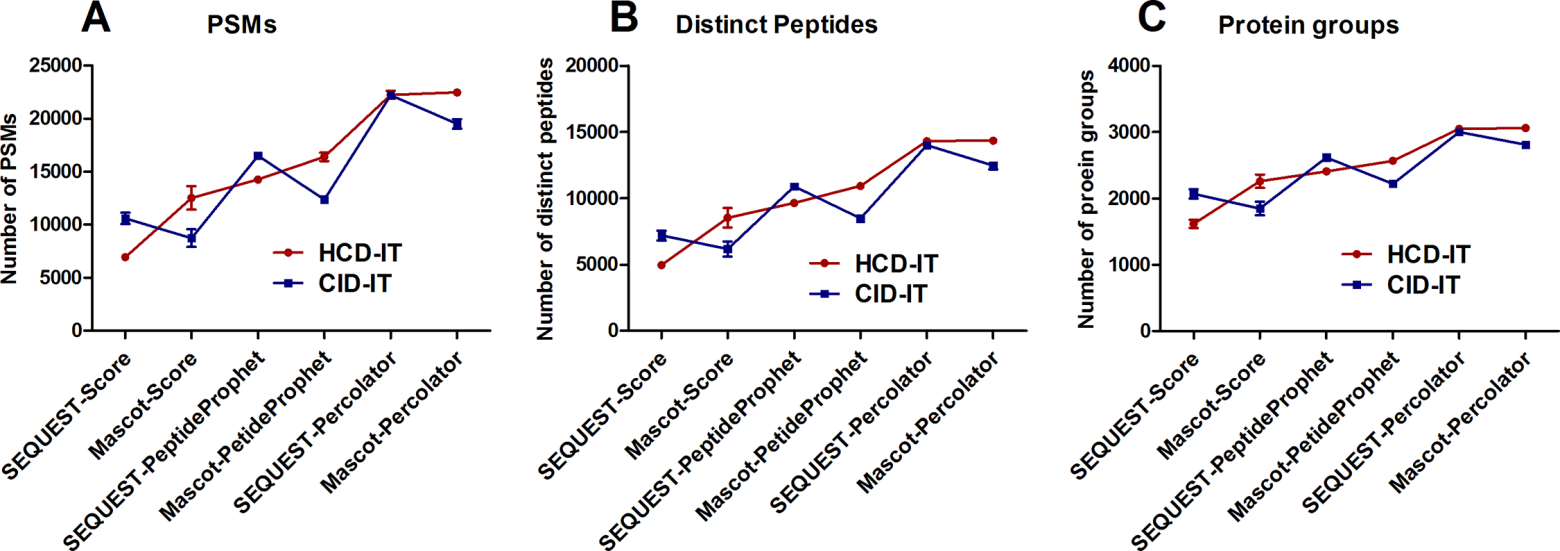
**(A) Peptide spectra matches (PSMs), (B) distinct peptides and (C) protein groups from the raw files for the comparison of HCD- and CID-type fragmentation using the back-to-back strategy (HCD-IT *vs*. CID-IT).** Six different MS data interpretation approaches were used in this study by combining SEQUEST or Mascot with different post-processing methods such as score-based, PeptideProphet, and Percolator.

To identify a relatively fair platform for peptide/protein identification, a total of six MS data interpretation approaches (search engines + post-processing methods) were used to analyze these raw files (Fig 1C). As shown in Fig 2, the CID-IT mode achieved significantly more PSMs (10597 vs. 6944), distinct peptides (7184 vs. 4964) and protein groups (2069 vs. 1620) than HCD-IT when using SEQUEST-Score or SEQUEST-PeptideProphet interpretation approaches. However, when using SEQUEST-Percolator for the data interpretation, similar quantities (PSMs, distinct peptides and protein groups) were provided by CID-IT and HCD-IT modes (Fig 2). Interestingly, opposite results were obtained by using Mascot coupled with the three data interpretation approaches. For instance, more PSMs (12531 vs. 8742), distinct peptides (8530 vs. 6169) and protein groups (2260 vs. 1849) were significantly achieved from the HCD-IT mode than the CID-IT mode using Mascot-Score (Fig 2). Therefore, the results of comparison between the HCD and CID modes were greatly biased by the MS data interpretation approaches. In this study, SEQUEST-Percolator and Mascot-Percolator obtained the highest number of quantities for the CID-IT and HCD-IT modes, respectiely. However, there was no significant difference (*p* > 0.05) between them as shown in S3A Fig. As expected, the overlap between CID-IT data (interpreted by SEQUEST-Percolator) and HCD-IT data (by Mascot-Percolator) from the same LC-MS replicate was excellent and more than 84.5% of peptides were co-identified (S3B Fig). We further analyzed the overlapped and unique peptides for each method, and the overlapped peptides tend to have higher identification scores and more amino acids as shown in S3C and S3D Fig.

**Fig 3:**
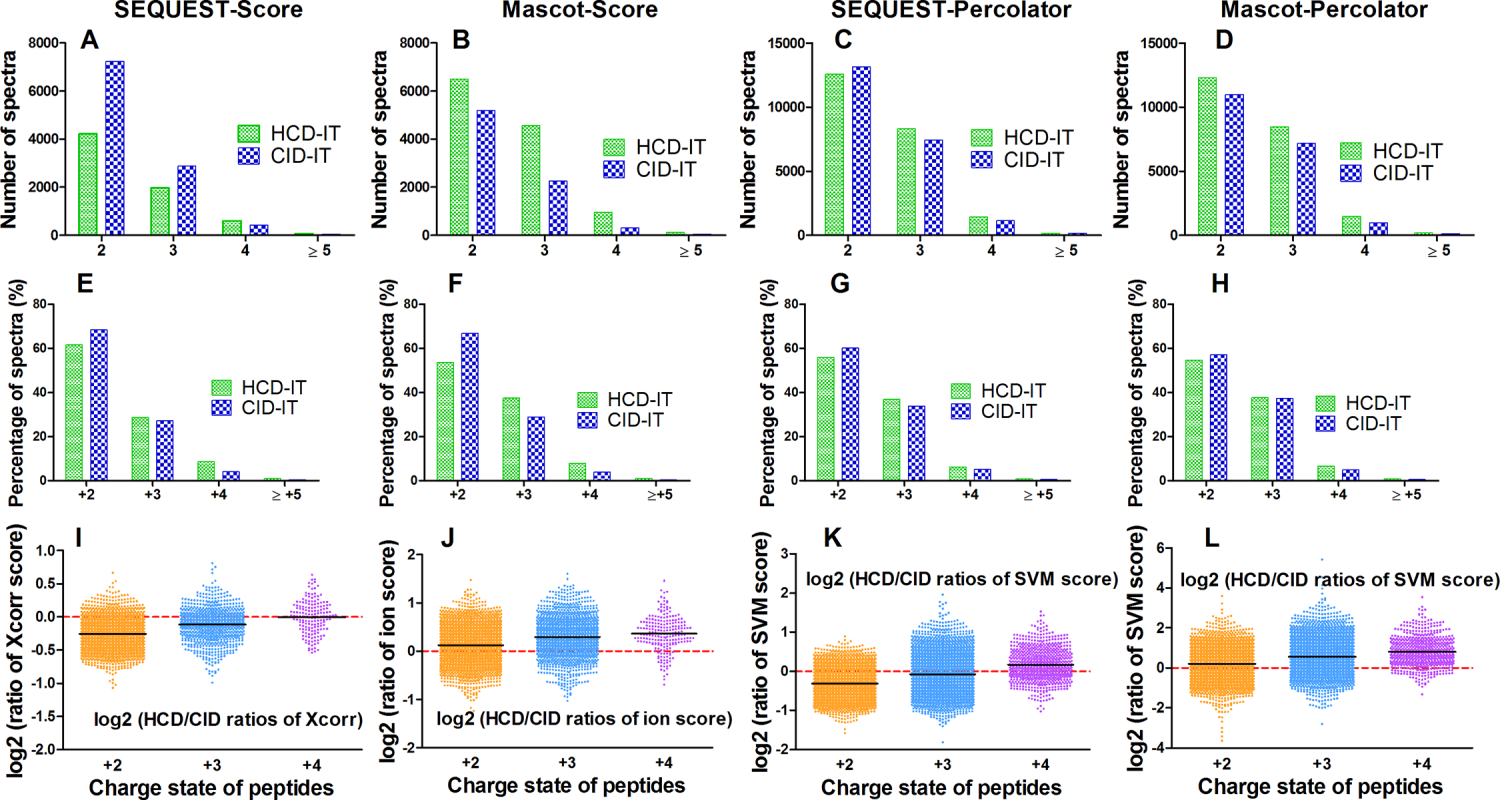
The comparison of HCD- and CID-type fragmentation using the back-to-back strategy (HCD-IT *vs*. CID-IT). (A-D) Number of spectra for +2, +3, +4 and ≥5 charged ions using different data interpretation methods. (E-H) Percentage of spectra for for +2, +3, +4 and ≥5 charged ions using different data interpretation methods. (I-L) Log2 (HCD/CID ratio of score) distributions for +2, +3 and +4 charged peptides. Xcorr for SEQUEST, ion score for Mascot, SVM score for Percolator related combinations were presented. One replicate of the back-to-back LC-MS analyses of HCD-IT versus CID-IT was randomly selected.

The distributions of peptide charge and ratio of searching score (i.e. Xcorr for SEQUEST, ion score for Mascot, and SVM score for Percolator) between HCD-IT and CID-IT for co-identified peptides were further investigated. One replicate of the back-to-back LC-MS analyses of HCD-IT *versus* CID-IT was randomly selected for this purpose. As shown in Fig 3A, 3B & 3D, more PSMs for each charge state in one type of fragmentation were identified when that type of fragmentation achieved more total PSMs by using the specific combination of search engine and post-processing method. For instance, Mascot-Score identified more PSMs (12531 vs. 8742) from HCD-IT than CID-IT (Fig 2), for each charge state more PSMs (i.e. +2 charged peptides, 6506 vs. 5190) were still got from HCD-IT than CID-IT (Fig 3B). Interestingly, though similar PSMs (or a little bit more PSMs from HCD-IT) were achieved from HCD-IT (22240) and CID-IT (22227) when using SEQUEST-Percolator (Fig 2), HCD-IT achieved fewer doubly charged (+2) PSMs (12556 vs. 13157) and more highly charged PSMs (≥ +3) (i.e. +3 charged peptides, 8313 vs. 7413) as shown in Fig 3C. The percentage distribution of different charged peptides further proved it. As shown in Fig 3E–3H, no matter how many PSMs were identified from HCD-IT or CID-IT by different interpretation approaches, HCD-IT achieved higher percentages of highly charged PSMs while CID-IT leant towards to doubly charged PSMs. We also investigated searching scores of peptides between HCD-IT and CID-IT. When using Mascot (coupled with score-based or Percolator), HCD-IT achieved higher search score than CID-IT (Fig 3J & 3L); for SEQUEST-Score or -Percolator, CID-IT achieved higher search score for doubly charged peptides and triply charged peptides but not quadruply charged peptides (Fig 3I & 3K).

In summary, the similar identifications including PSMs, distinct peptides and proteins groups were achieved from HCD-IT (Mascot- or SEQUEST-Percolator) and CID-IT (SEQUEST-Percolator) when using the relatively fair platform of MS data interpretation for each MS2 acquisition method. These results indicated that HCD- and CID-type MS2 fragmentation on Orbitrap Fusion mass spectrometer may contribute almost equally for the peptide/protein identification. Moreover, the percentage of highly charged peptides appeared higher in HCD-IT data than that in CID-IT.

### Back-to-back comparison for OT- and IT-type MS2 detections

**Fig 4:**
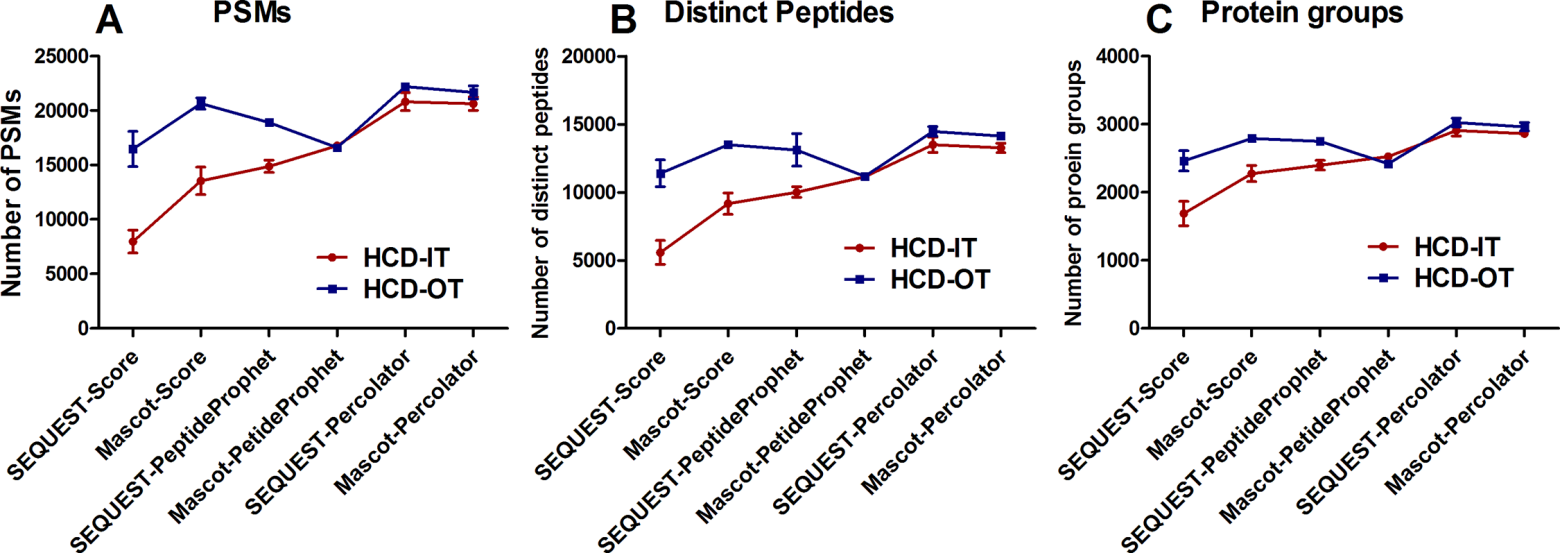
**(A) Peptide spectra matches (PSMs), (B) distinct peptides and (C) protein groups from the raw files for the comparison of OT- and IT-type detection using the back-to-back strategy (HCD-IT *vs*. HCD-OT).** Six different MS data interpretation approaches were used in this study by combining SEQUEST or Mascot with different post-processing methods such as score-based, PeptideProphet, and Percolator.

MS data interpretation approaches described above were also applied to the back-to-back analyses of HCD-IT *versus* HCD-OT to perform comparison between OT and IT detections. As shown in Fig 4, Percolator also achieved higher identifications than score-based and PeptideProphet methods, which was search engine-independent. More PSMs, distinct peptides and protein groups were identified from MS data generated in HCD-OT mode than HCD-IT mode when the data was analyzed with selected MS data interpretation approaches except Mascot-PeptideProphet (Fig 4A–4C). For instance, 20659 PSMs, 13496 distinct peptides and 2791 protein groups were identified in the HCD-OT mode using Mascot-Score, while only 13549 PSMs, 9173 distinct peptides and 2275 protein groups in the HCD-IT mode. In this study, SEQUEST-Percolator achieved the highest number of identifications for both HCD-OT and HCD-IT modes. Though the difference between HCD-IT and HCD-OT by SEQUEST-Percolator was not significant (*p* >0.05), HCD-OT obtained a slightly higher number of identifications than HCD-IT (Fig 4). However, the advantage of speed for IT detection was not fully utilized here because of the back-to-back strategy, while the advantage of accuracy for OT still contributes to improve identification.

**Fig 5:**
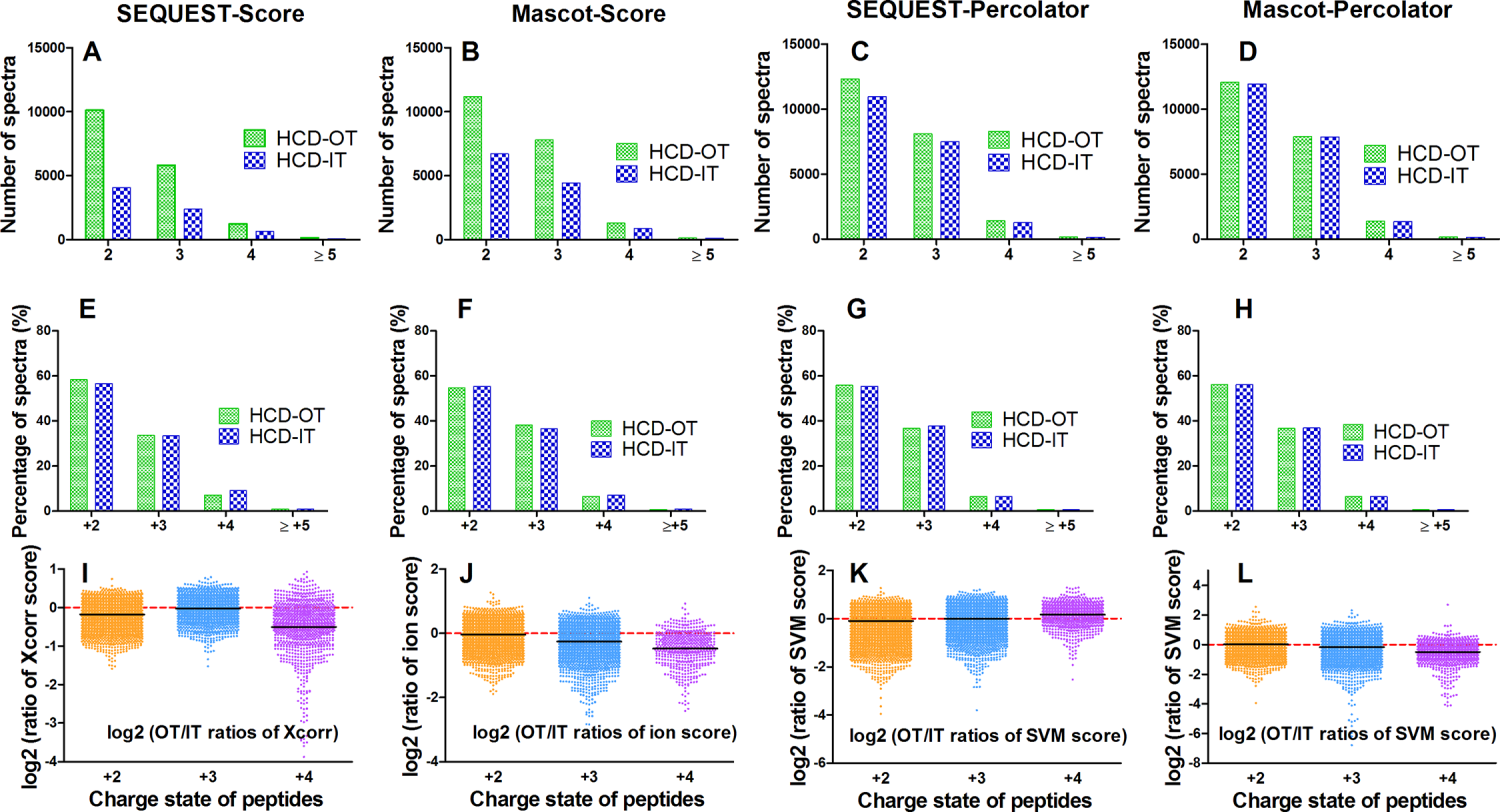
The comparison of OT- and IT-type detection using the back-to-back strategy (HCD-IT *vs*. HCD-OT). (A-D) Number of spectra for +2, +3, +4 and ≥5 charged ions using different data interpretation methods. (E-H) Percentage of spectra for for +2, +3, +4 and ≥5 charged ions using different data interpretation methods. (I-L) Log2 (OT/IT ratio of score) distributions for +2, +3 and +4 charged peptides. Xcorr for SEQUEST, ion score for Mascot, SVM score for Percolator related combinations were presented. One replicate of the back-to-back LC-MS analyses of HCD-IT versus HCD-OT was randomly selected.

The distributions of peptide charge and ratio of searching scores between HCD-IT and HCD-OT for co-identified peptides were also investigated. One replicate of the back-to-back LC-MS analyses of HCD-IT *versus* HCD-OT was randomly selected. As shown in Fig 5A–5D, because of the fact that HCD-OT identified more total PSMs than HCD-IT (Fig 4), more PSMs for each charge state were still obtained in HCD-OT mode than HCD-IT mode. Different from the results of HCD-IT/CID-IT comparison described above, the percentage distributions of PSMs identified by HCD-IT and HCD-OT were similar, though the numbers were significantly different because of the use of different interpretation approaches (Fig 5E–5H). For instance, more PSMs (20659 vs. 13549) were identified from HCD-OT than HCD-IT using Mascot-Score, but the percentages of different charged peptides were similar between HCD-OT and HCD-IT (54.7% vs. 55.4% for +2 charged peptides, 38.2% vs 36.6% for +3) as shown in Fig 5F. Therefore, the use of OT- and IT-type detection did not affect the identification of peptides with different charge state. For the searching score, since the scoring systems of SEQUEST and Mascot are not optimized for high-resolution MS2 spectra, the scores from these two engines turned out to be higher for peptides from IT than those from OT, and thus the majority of log2 transformed OT/IT ratios of scores were below zero (Fig 5I & 5J). Percolator, on the other hand, has considered many features including the accuracy of MS2 spectra[22], which improves the scoring system of these two search engines and more similar scores were given for the peptides co-identified by HCD-OT and HCD-IT as expected (Fig 5K & 5L).

In summary, a slight increase of identifications were achieved from HCD-OT than HCD-IT in the back-to-back LC-MS analyses when SEQUEST-Percolator, a relatively fair platform, was used for MS data interpretation. Moreover, lower SEQUEST-Xcorr or Mascot-ion scores were achieved for the peptides analyzed in the OT mode than that in the IT mode because of the sub-optimized scoring system. It is important to note that, because of the different mechanisms of ion detection by OT and IT, higher AGC target and maximum injection were used for OT than IT in routine analysis.

### Evaluating different MS2 acquisition strategies: HCD-IT, HCD-OT, CID-IT, and CID-OT

**Fig 6:**
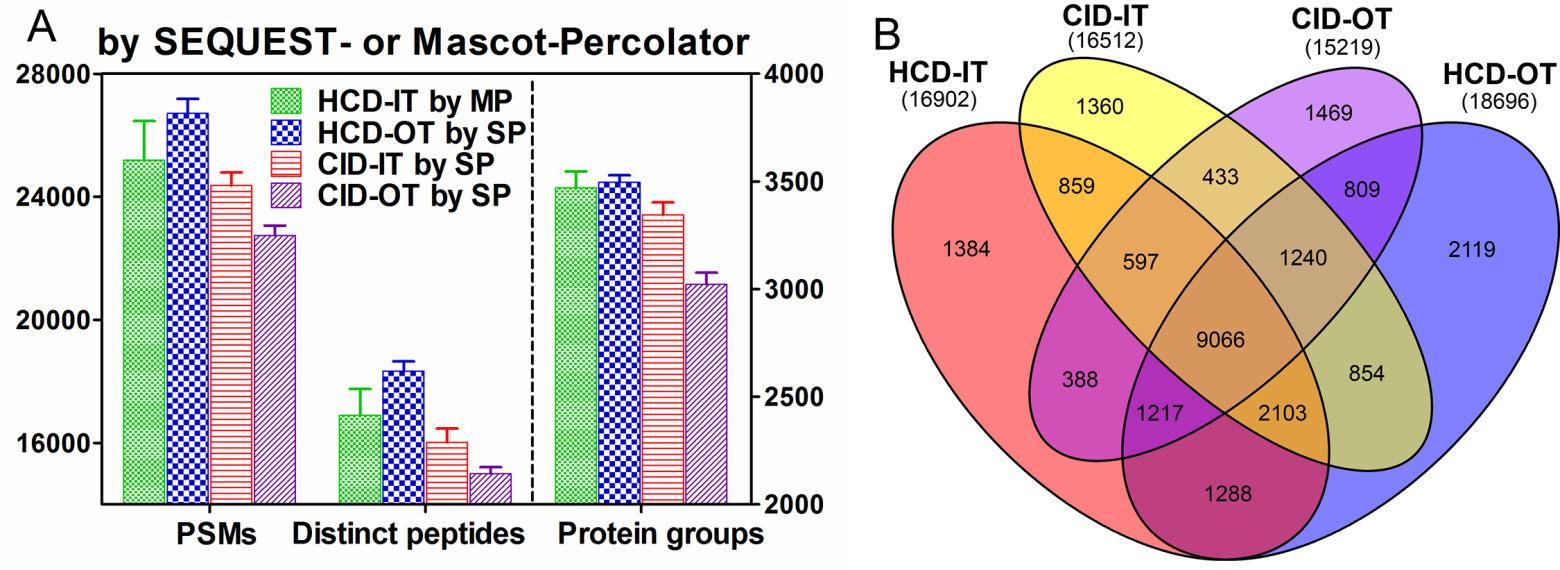
Comparison of four MS2 acquisition methods: HCD-OT, HCD-IT, CID-IT and CID-OT in Orbitrap Fusing using suitable data interpretation approaches. (A) Peptide spectra matches (PSMs), distinct peptides and protein groups from separate analyses of human cell proteome. (B) The unique peptide overlaps between these four MS2 acquisition methods. MP: Mascot-Percolator; SP: SEQUEST-Percolator.

To further address which combination of fragmentation and detection would give the best performance, the human cell proteome (PANC-1 cells) was also used to evaluate different MS2 strategies in Orbitrap Fusion. Four MS2 acquisition methods were theoretically available in Orbitrap Fusion as shown in S2 Fig. Of these four methods, HCD-IT, HCD-OT, and CID-IT were commonly used and applied in various proteomics studies. Here three replicates for each of them were alternatively analyzed. As shown in S4 Fig, except Mascot-PeptideProphet, HCD-OT achieved the best performance in PSMs, distinct peptides and protein groups in other five combinations. More IDs were given when analyzing HCD-IT by Mascot than SEQUEST; on the contrary, more IDs were given when analyzing CID-IT by SEQUEST than Mascot as described above, no matter which post-processing approach was used. To perform the relatively fair comparison between these three MS2 pipelines, the best interpretation methods for each pipeline were selected. Here HCD-OT and CID-IT by SEQUEST-Percolator (SP) and HCD-IT by Mascot-Percolator (MP) were analyzed. As shown in Fig 6A, these three MS2 pipelines achieved similar results in PSMs, distinct peptides and protein groups. Statistical analysis between any two of them showed that significant difference (p < 0.05) was only observed between HCD-OT (an average of 26708 PSMs, 18332 distinct peptides, 3495 protein groups) and CID-IT (24367, 16022, 3346). For HCD-IT by MP, an average of 25192 PSMs, 16901 distinct peptides and 3471 protein groups were observed. Moreover, the file size of HCD-OT here is around 1.4 gigabyte (GB), smaller than HCD-IT (~3.1 GB) and CID-IT (~2.8 GB). All the MS/MS data is collected in the centroid mode. The smaller file size of HCD-OT enabled multiplex quantitative analyses (i.e. 40-plex) for algorithms such as Thermo SIEVE and reduced the amount of time of identification and quantification procedures. Therefore, for Orbitrap Fusion, the MS2 pipelines of HCD-OT were suggested as the first choice for identification and multiplex quantification analysis.

In addition, the MS2 acquisition method of CID-OT is theoretically available on Fusion Orbitrap, though it has not been discussed in previous studies. In this study three replicates of LC-MS/MS runs by the CID-OT pipeline were performed by using the same LC column and peptide mixture. Because of the comparable IDs from HCD-IT and CID-IT, the similar result may be achieved in the comparison of HCD-OT and CID-OT if the efficiency of ion transfer is still similar between them. However, CID-OT, which performs the fragmentation in CID and MS2 detection in Orbitrap, is not an effective MS2 acquisition method because of the long distance between the fragmentation cell and Orbitrap as shown in S2 Fig. Surprisingly, CID-OT achieved more than 3,000 protein groups (by SEQUEST-Percolator) from one-shot LC-MS/MS run (Fig 6A), only a decrease of 10%-14% than other three MS2 pipelines.

No significant difference (*p* >0.05) were observed between CID-IT and HCD-IT (Fig 6A), consistent with the results from the back-to-back analyses. To further validate the finding described above that more highly charged peptides were achieved from HCD than CID, the charge state of peptides were further analyzed on the separate LC-MS/MS runs here. The respective runs with highest identifications for each method were selected for comparison. As shown in S5A Fig, since HCD-OT achieved more total identifications than HCD-IT and CID-IT, more identifications at each charge state were also observed. However, the percentage of doubly charged peptides from HCD-OT or HCD-IT was still lower than that from CID-IT (S5B Fig), agree well with the results from the back-to-back analyzes above.

From our results described above, the significantly different results will be achieved when analyzing the same CID-IT data with SEQUEST and Mascot. This may explain the divergent conclusions recently reported by Mann’s group[7] and Gygi’s group[2] who used different search engines and post-processing algorithms to evaluate HCD- and CID-type fragmentation (actually HCD-OT versus CID-IT). Though the same dataset from Mann’s group was individually analyzed by these two groups, the opposite conclusions were achieved. SEQUEST- linear discriminant analysis (LDA) and MaxQuant (Mascot v2.2) were respectively used in Gygi’s group and Mann’s group. PeptideProphet originally uses the LDA classifier to separate correct and incorrect PSMs in an unsupervised fashion (i.e., without decoy information)[17] and is improved by the semi-supervised approach using decoy PSMs to estimate probabilities from the discriminant scores[24]. Therefore, the MS data interpretation methods they used are respectively similar as SEQUEST-PeptideProphet and Mascot-Score we tested here. In this study similar IDs were achieved from HCD-OT and CID-IT analysis of the PANC-1 cell sample when using SEQUEST-PeptideProphet (S4B Fig), but significantly more PSMs (~3-fold) were achieved from HCD-OT than CID-IT (S4D Fig) using Mascot-Score. Because of this fact, it is not surprising that different conclusions could be made from the same dataset when using different interpretation methods. Therefore, to achieve a fair comparison, respective optimal data interpretation methods were required when performing comparison between different MS2 techniques. In this study, Percolator associated MS data interpretation methods were suggested based on our results.

### Overlaps between different MS2 pipelines

Further investigation of peptide identification overlap between HCD-IT, HCD-OT, CID-IT and CID-OT was performed by using the results from SEQUEST-Percolator. The raw files with highest identifications by SEQUEST-Percolator for each MS2 acquisition method were selected. As shown in Fig 6B, 15219, 16512, 16902 and 18696 distinct peptides were respectively identified from data generated by CID-OT, CID-IT, HCD-IT and HCD-OT. A total of 25186 unique peptides were detected from these four raw files, and 71%-77% overlaps (double shared peptides over the sum of peptides) were observed between any two of them. The highest number of unique peptides (21945) was achieved from the combination of HCD-OT and CID-IT among the combinations of any two of them (S6A Fig). Moreover, we further investigated the overlaps between two technical replicates using these four MS2 strategies respectively. As shown in S6B Fig, slight higher overlaps (80%-83%) between technical replicates were observed. The highest number of unique peptides (22169) is from the combination of two HCD-OT replicates with an overlap of 80%. Therefore, the combination of different MS2 pipelines (i.e. HCD-OT & CID-IT) may be not necessary for identification here.

## Conclusions

Here we have investigated multiple MS2 acquisition methods on the Orbitrap Fusion, a mass spectrometry that incorporates quadrupole, Orbitrap, and dual-cell ion trap analyzers and enables to enhance proteome coverage and peptide identification rates. Percolator associated data interpretation approach was selected as a fair platform to perform the respective comparisons of different methods of fragmentation, detection, and MS2 acquisition. Different charged peptides were observed to be preferred to CID or HCD fragmentation events. HCD-OT and HCD-IT these two MS2 acquisition methods were suggested for peptide identification using Orbitrap Fusion. Further MS2 acquisition methods, such as ETD-OT, ETD-IT, can also be incorporated and applied for highly charged peptides. Moreover, the combination of CID for doubly charged peptides and HCD for highly charged peptides is possible when both of their collision cells are close to the Orbitrap analyzer or with respective ones, or the same precursor derived fragmentation ions from CID and HCD are stored in the same cell and then detected together. All these strategies are possible in principle and improvements in mass spectrometry, data interpretation techniques, and clinical application are interesting for the proteomics community.

## Supporting Information

S1 Fig Evaluations of some parameters for MS instrument and database search. (PDF)

S2 Fig The four MS2 acquisition methods in the Orbitrap Fusion mass spectrometry analyzed in this study. (PDF)

S3 Fig The comparison between HCD-IT by MP and CID-IT by SP for separate LC-MS/MS analyses (N = 3). (PDF)

S4 Fig Peptide spectra matches (PSMs), distinct peptides and protein groups from the separate LC-MS/MS analyses for the comparison of HCD-OT, HCD-IT, and CID-IT. (PDF)

S5 Fig The comparison of HCD-OT by SP, HCD-IT by MP, and CID-IT by SP for separate LC-MS/MS analyses. (PDF)

S6 Fig Respective overlaps between (A) different MS2 acquisition methods and (B) duplicate analyses of each MS2 acquisition methods.
(PDF)
