# Supplementary material for: Performance Investigation of Proteomic Identification by HCD/CID Fragmentations in Combination with High/Low-Resolution Detectors on a Tribrid, High-Field Orbitrap Instrument
Source: PLoS One. 2016 Jul 29;11(7):e0160160. doi: 10.1371/journal.pone.0160160 (PMC4966894; doi:10.1371/journal.pone.0160160)
Supplement: S1 Fig — (PDF) [file pone.0160160.s001.pdf]

S1 Fig

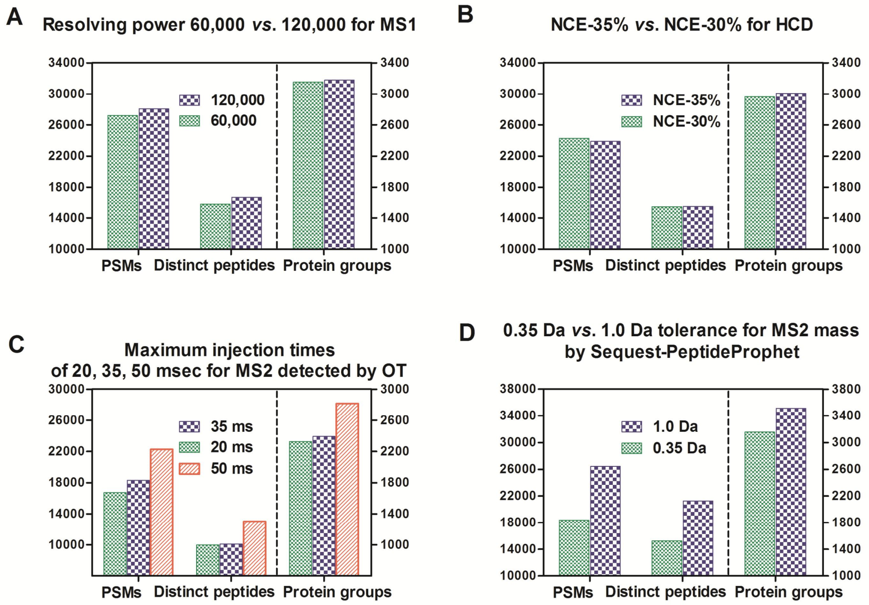

S1 Fig. Evaluations of some parameters for MS instrument and database search. Investigations of (A) different resolutions for MS1 analysis by Orbitrap, (B) different normalized collision energy (NCE) for HCD, (C) different maximum injection times for MS2 analysis by Orbitrap, and (D) different tolerances for MS2 mass while performing database search by Sequest-PeptideProphet. Human cell sample (PANC-1) was used for A, B and C. The MS data of yeast by HCD-IT analysis of Orbitrap Fusion was downloaded from ChorusProject.org under project name SignleShot_Fusion.
