# Supplementary material for: Performance Investigation of Proteomic Identification by HCD/CID Fragmentations in Combination with High/Low-Resolution Detectors on a Tribrid, High-Field Orbitrap Instrument
Source: PLoS One. 2016 Jul 29;11(7):e0160160. doi: 10.1371/journal.pone.0160160 (PMC4966894; doi:10.1371/journal.pone.0160160)
Supplement: S2 Fig — (PDF) [file pone.0160160.s002.pdf]

S2 Fig

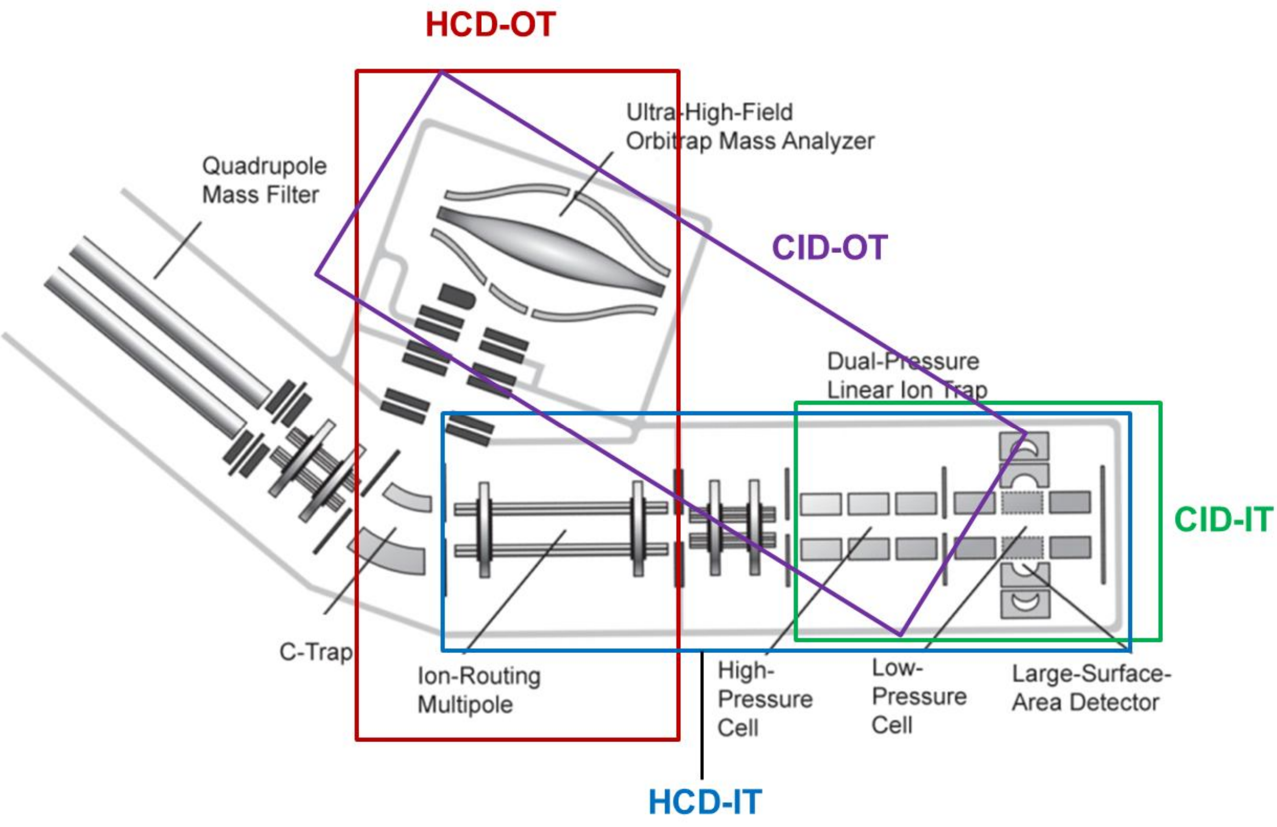

S2 Fig. The four MS2 acquisition methods in the Orbitrap Fusion mass spectrometry analyzed in this study. HCD-OT, HCD-IT, CID-IT and CID-OT were indicated here. ETD-IT and ETD-OT are also available in Orbitrap Fusion, but they are not investigated in this study.
