# Supplementary material for: Performance Investigation of Proteomic Identification by HCD/CID Fragmentations in Combination with High/Low-Resolution Detectors on a Tribrid, High-Field Orbitrap Instrument
Source: PLoS One. 2016 Jul 29;11(7):e0160160. doi: 10.1371/journal.pone.0160160 (PMC4966894; doi:10.1371/journal.pone.0160160)
Supplement: S3 Fig — (PDF) [file pone.0160160.s003.pdf]

S3 Fig

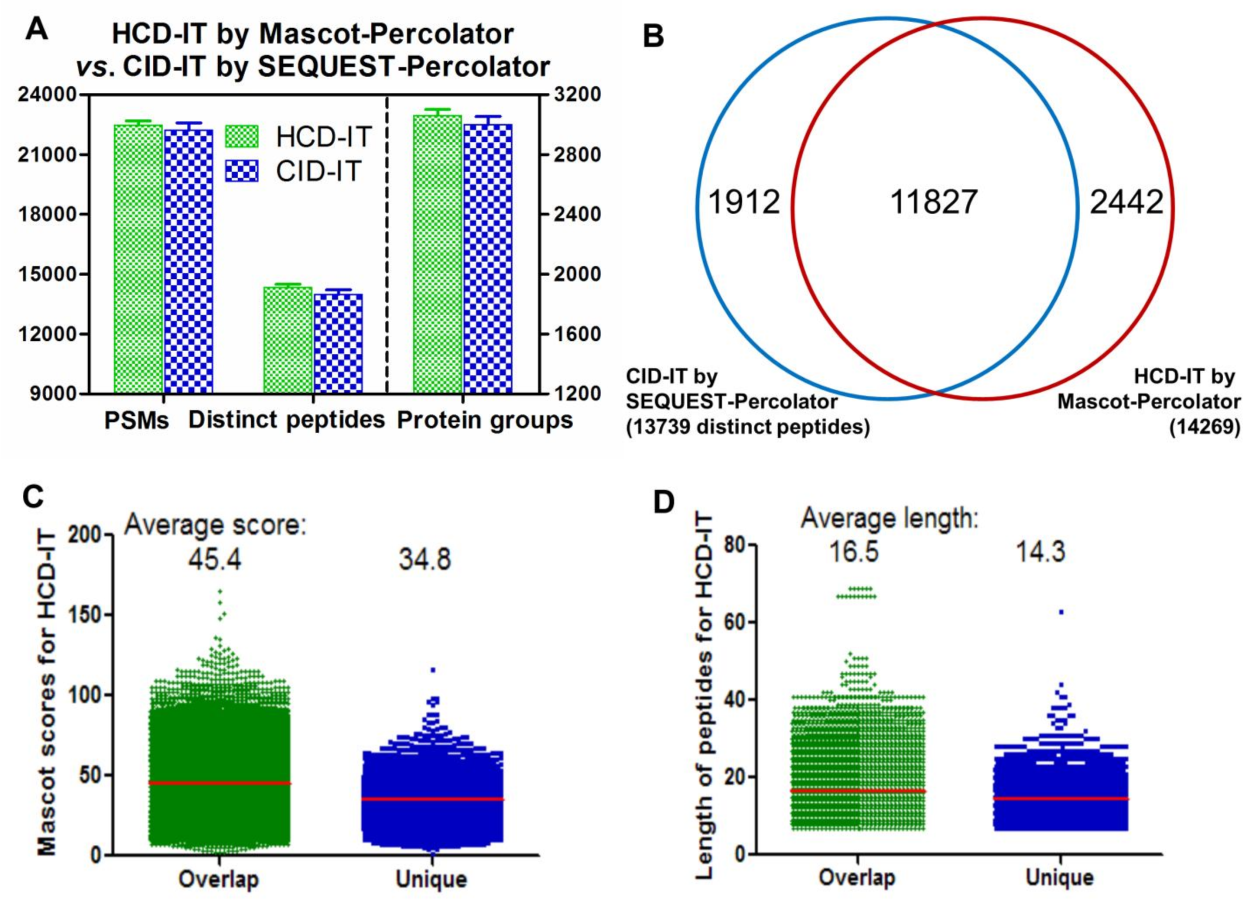

S3 Fig. The comparison between HCD-IT by MP and CID-IT by SP for separate LC-MS/MS analyses (N=3). (A) Peptide spectra matches (PSMs), distinct peptides and protein groups for the comparison of HCD-IT by MP and CID-IT by SP. (B) Overlap of distinct peptides between HCD-IT by MP and CID-IT by SP. (C) The Mascot scores in overlapped peptides and unique peptides of HCD-IT. (D) The peptide lengths in overlapped peptides and unique peptides of HCD-IT. The LC-MS/MS run providing the highest number of distinct peptides were selected for each type. SP: SEQUEST-Percolator; MP: Mascot-Percolator.
