# Supplementary material for: Performance Investigation of Proteomic Identification by HCD/CID Fragmentations in Combination with High/Low-Resolution Detectors on a Tribrid, High-Field Orbitrap Instrument
Source: PLoS One. 2016 Jul 29;11(7):e0160160. doi: 10.1371/journal.pone.0160160 (PMC4966894; doi:10.1371/journal.pone.0160160)
Supplement: S4 Fig — (PDF) [file pone.0160160.s004.pdf]

S4 Fig

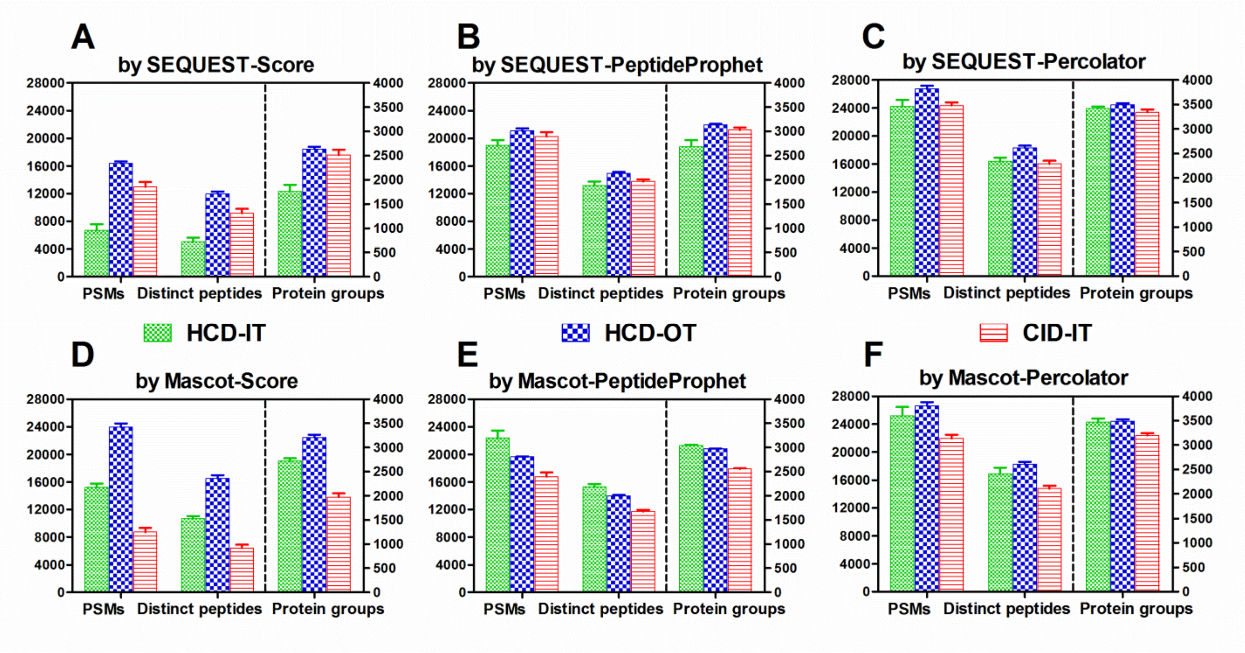

S4 Fig. Peptide spectra matches (PSMs), distinct peptides and protein groups from the separate LC-MS/MS analyses for the comparison of HCD-OT, HCD-IT, and CID-IT. Six different MS data interpretation approaches were used in this study (A-F) by combining SEQUEST or Mascot with different post-processing methods such as score-based, PeptideProphet, and Percolator.
