# Supplementary material for: Performance Investigation of Proteomic Identification by HCD/CID Fragmentations in Combination with High/Low-Resolution Detectors on a Tribrid, High-Field Orbitrap Instrument
Source: PLoS One. 2016 Jul 29;11(7):e0160160. doi: 10.1371/journal.pone.0160160 (PMC4966894; doi:10.1371/journal.pone.0160160)
Supplement: S5 Fig — (PDF) [file pone.0160160.s005.pdf]

S5 Fig

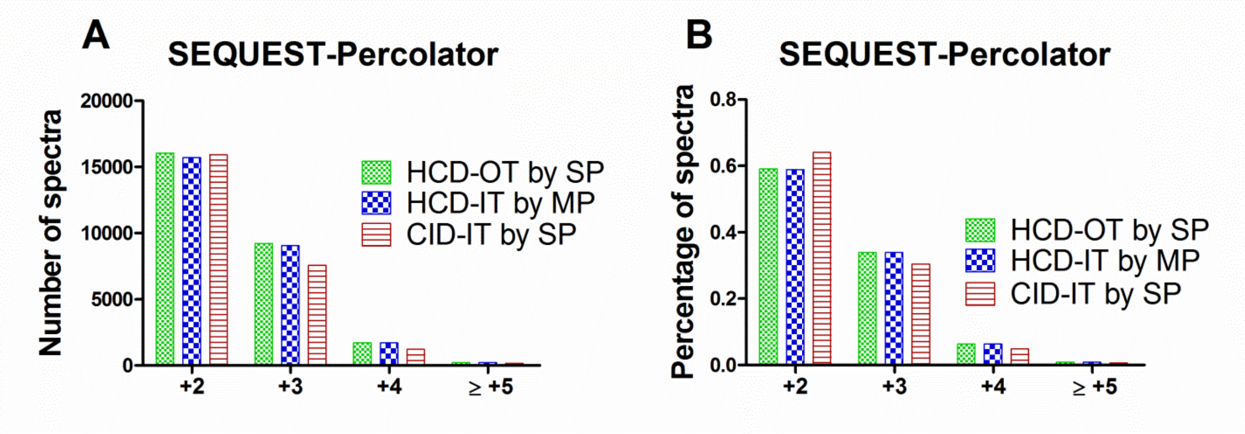

S5 Fig. The comparison of HCD-OT by SP, HCD-IT by MP, and CID-IT by SP for separate LC-MS/MS analyses. (A) Number of spectra for +2, +3, +4 and ≥5 charged; (B) Percentage of spectra for for +2, +3, +4 and ≥5 charged ions from these three MS2 acquisition methods. SP: SEQUEST-Percolator; MP: Mascot-Percolator.
