# Supplementary material for: Performance Investigation of Proteomic Identification by HCD/CID Fragmentations in Combination with High/Low-Resolution Detectors on a Tribrid, High-Field Orbitrap Instrument
Source: PLoS One. 2016 Jul 29;11(7):e0160160. doi: 10.1371/journal.pone.0160160 (PMC4966894; doi:10.1371/journal.pone.0160160)
Supplement: S6 Fig — Respective overlaps between (A) different MS2 acquisition methods and (B) duplicate analyses of each MS2 acquisition methods. (PDF) [file pone.0160160.s006.pdf]

S6 Fig

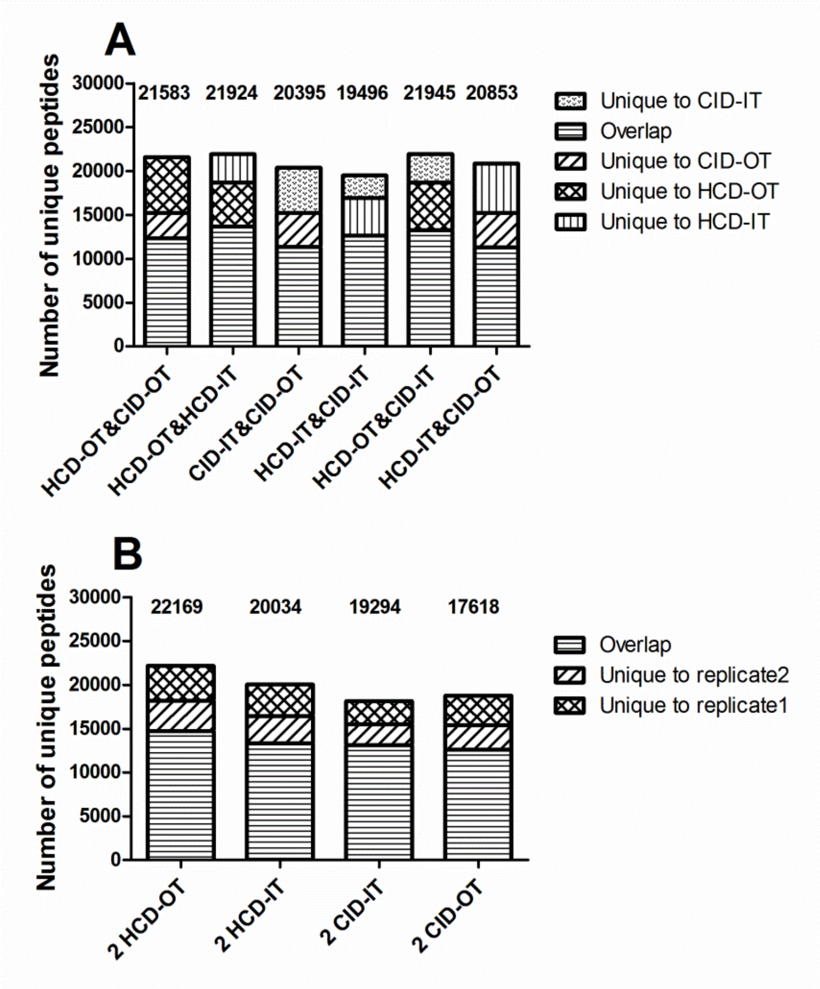

S6 Fig. Respective overlaps between (A) different MS2 acquisition methods and (B) duplicate analyses of each MS2 acquisition methods. The overlaps between duplicate analyses of each MS2 acquisition methods are obviously higher than that between different MS2 acquisition methods. However, the highest number of unique peptides is achieved from duplicate analyses of HCD-OT, followed by combination of HCD-OT and CID-IT. Here the data interpretation SEQUEST-Percolator was used for these raw files.
